# The Duty of the Physician to His Patient

**Published:** 1894-05

**Authors:** W. H. Elliott

**Affiliations:** Savannah, Ga.


ATLANTA

### MEDICAL AND SURGICAL JOURNAL.

VOL. XI. MAY, 1894. No. 3.

EDITED BY

LUTHER B. GRANDY, M.D., AND MILLER B. HUTCHINS, M.D.,

WITH THE CO-OPERATION OF

H. V. M. MILLER, M.D., LL.D., VIRGIL O. HARDON, M.D.,
FLOYD W. McRAE, M.D., ARTHUR G. HOBBS, M.D.,
AND H. R. SLACK, M.D., PH.M.

### ORIGINAL COMMUNICATIONS.

### THE DUTY OF THE PHYSICIAN TO HIS PATIENT.*

BY W. H. ELLIOTT, M.D.,
SAVANNAH, GA.

Much of what I shall say is of record and is, or should be, known to you. Yet I think it will be well, not only to refresh your memories by a recital of the law, but to ask your attention to some practical applications of its precepts.

First, then, for the letter of the law. What are the obligations it implies upon the physician? I read the following from the American and English Encyclopedia of Law:

"Physicians and surgeons, by holding themselves out to the world as such, impliedly contract that they possess the reasonable and ordinary qualifications of their profession, and are under a duty to exercise reasonable and ordinary care, skill and diligence: but that is the extent of their liability."

In the words of Justice Tyndall, "Whoever undertakes to prac-

*The President's Address delivered before the Georgia Medical Association, Atlanta, April 18, 1894.

tice any art or profession assumes an obligation both civil and professional, which, though implied, has all the force and validity of a formal contract."

"The reasonable and ordinary care, skill and diligence that the law requires of physicians and surgeons is such as physicians in the same general neighborhood, in the same general line of practice, ordinarily have and exercise in like cases."

"A different rule has been held in Pennsylvania. In two cases the court held that such skill was required as thoroughly educated surgeons ordinarily employ, but the weight of authority is as above stated."

"The true measure is that ordinarily exercised in the profession by the members thereof as a body: that is, the *average* of the reasonable skill and diligence ordinarily exercised by the profession as a whole; not that exercised by the *thoroughly educated;* nor yet that exercised by the *moderately educated,* nor merely of the well educated, but the *average* of the thorough, the well, and the moderate."

"The locality in which a physician or surgeon practices is to be taken into account. One who lives in a large city and enjoys the opportunities of metropolitan schools and hospitals is justly held to a higher measure of accountability than one who could not have such advantages."

But, "Physicians and surgeons should keep abreast of the times and make use of the latest and most improved methods and appliances, having regard to the locality and general practice of the profession, and it is for the jury to decide from the particular circumstances of each case whether the physician or surgeon has fulfilled his duty in this respect."

"A departure from approved methods of practice, resulting in injury to the patient, will render the practitioner liable, however honest the intention and expectation of benefit to the patient may be."

"If a physician or surgeon be sent for to attend a patient, the effect of his responding to the call, in the absence of any special agreement, will be an engagement to attend to the case as long as it requires attention, unless he gives notice of his intention to discontinue his visits, or is dismissed by the patient; and he is bound to exercise a reasonable and ordinary care and skill in determining when his attendance should cease." He is also bound, in dismissing a case to give his patient such advice in regard to his mode of life and the proper care of himself as the circumstances of the case may require.

"Physicians and surgeons are bound to give their patient the benefit of their best judgment, but they are not liable for a mere error of judgment. An error of judgment may be so gross, however, as to be inconsistent with reasonable care, skill and diligence."

"There is no implied warranty on the part of the physician that he will effect a cure; and he can be held as a guarantor of success, only in virtue of an express agreement. The mere failure to effect a cure raises no presumption of a want of proper care, skill and diligence.

"If a physician or surgeon is not competent, or feels that he is not competent, to treat a case, it is his duty to recommend the employment of another, but if he is competent and so considers himself, and is in doubt concerning the case, he should use his best judgment as to consultation with other physicians or surgeons. The refusal to accept the assistance of another in the treatment of a case imposes no higher duty upon a physician or a surgeon."

"The fact that a physician or surgeon renders services gratuitously does not affect his duty to exercise reasonable and ordinary care, skill and diligence."

"If the fault or negligence of a patient or his attendant contributes to the patient's injury, he cannot recover for malpractice by the physician or surgeon, and this is true in a case where the natural temperament or physicial weakness of the patient are the contributing causes of injury."

"Where the injury attributable to the fault of the patient can be separated from that of the physician, the former may recover for so much of the injury as is due to the fault of the latter."

"When a patient relying on his own judgment, gives directions as to his treatment he cannot claim damages of the physician for the consequences."

"The burden of proof to show contributory negligence is upon the defendant in an action for malpractice."

These are the principles which the law lays down for the medical profession in general: Competency first, then diligence.

For the surgeon, there are some obligations peculiar to his branch of the profession.* The responsibility for the success of every operation which he performs is individual. He may decline to undertake any case, but, having accepted the trust, he alone is responsible for the results of treatment. Neither the attending physician nor the consulting surgeon assumes any portion of the obligation. Not only during the operation, but throughout the entire treatment, the conduct of the surgeon must be characterized by fidelity to the patient, and a uniform and consistent application of skill in the management of the case.

Failure at any time to meet the ordinary indications in the case vitiates the entire attendance, for the obligation is continuous to the termination.

In view of these facts it is important that the surgeon should make every case which he undertakes peculiarly his own. He should forecast every possible source of failure and be prepared for every possible emergency; for he is most ready to take responsibilities and to bear them easily, who can best estimate what are the risks and difficulties which he is to incur.

This individual responsibility is not recognized by the Code of Ethics, but to show that the mind of the profession is disposed to admit the correctness of this view of the question, I quote from the report of the committee on amending the Code, made at the last meeting of the American Medical Association:

"We recommend the more accurate definition of the term 'consultation,' as we find good reason to believe that serious estrangement has arisen between physicians because of the different ideas they attached to this term. The Code of Ethics says, 'that in a consultation the responsibility must be equally divided between the medical attendants; they must equally share the credit as well as the blame of failure.'

"With this statement before us, it is clear that there can be no consultation when one physician meets another for the purpose of obtaining from him an account of the case or pertinent facts of

* Stephen Smith, *Operative Surgery*.

family history, or a record of the past management of the case, in order that he may more intelligently assume the entire responsibility of its future conduct."

From this data it is clear that usually the specialist does not consult with the general practitioner. He simply obtains all the facts the general practitioner possesses preparatory to assuming full control of the case."

The principles of aseptic and antiseptic surgery, having been accepted by the profession, impose new obligations upon the surgeon. If he fails to apply these principles he can justly be held responsible for failure.

As all the ordinary operations are thus rendered more safe, the prognosis in all cases requiring operative procedure is more favorable and the field of operative surgery has been much enlarged. Many operations can now be undertaken which before were unjustifiable. The surgeon is bound to give the patient the benefit of all the advantages which have been gained by this advance in the science of surgery.

Thus speaks justice; cold and hard perhaps, but drawing the line of the equities straightly between the physician and the patient.

Now turn we to the Code of Medical Ethics of the American Medical Association. This Code is formally adopted by the Constitution of the Medical Association of Georgia, and the fact of our membership makes it binding upon us. It is an obligation voluntarily assumed, and, therefore, we are bound by the highest dictates of honor to faithfully observe its injunctions.

But what says the Code of the physician's duty to his patients?

"A physician should not only be ever ready to obey the calls of the sick, but his mind ought also to be imbued with the greatness of his mission, and the responsibility he habitually incurs in its discharge. *These obligations are the more deep and enduring, because there is no tribunal, other than his own conscience, to adjudge penalties for carelessness or neglect.* Physicians should, therefore, minister to the sick with due impressions of the importance of their office; reflecting that the ease, the health and lives of those committed to their charge depend on their skill, attention and fidelity. They should study, also, in their deportment, so to unite tenderness with firmness, and condescension with authority, as to inspire the minds of their patients with gratitude, respect and confidence.

"Every case committed to the charge of a physician should be treated with attention, steadiness and humanity. Reasonable indulgence should be granted to the mental imbecility and caprices of the sick. Secrecy and delicacy, when required by peculiar circumstances, should be strictly observed, and the familiar and confidential intercourse to which physicians are admitted in their professional visits, should be used with discretion, and the most scrupulous regard to fidelity and honor. The obligation of secrecy extends beyond the period of professional service; none of the privacies of personal and domestic life, no infirmity of disposition or flaw of character, observed during professional attendance, should ever be divulged by the physician, except when he is imperatively required to do so. The force and necessity of this obligation are indeed so great that professional men have, under certain circumstances, been protected in their observance of secrecy by courts of justice."

"Frequent visits to the sick are in general requisite, since they enable the physician to arrive at a more perfect knowledge of the disease, to meet promptly every change which may occur, and also tend to preserve the confidence of the patient. But unnecessary visits are to be avoided, as they give useless anxiety to the patient, tend to diminish the authority of the physician and render him liable to be suspected of interested motives."

"A physician should not be forward to make gloomy prognostications, because they savor of empiricism by magnifying the importance of his services in the treatment or cure of the disease. But he should not fail, on proper occasions, to give to the friends of the patient timely notice of danger when it really occurs; and even to the patient himself, if absolutely necessary. This office, however, is so peculiarly alarming when executed by him that it ought to be declined whenever it can be assigned to any other person of sufficient judgment and delicacy, for the physician should be the minister of hope and comfort to the sick; that by such cordials to the drooping spirit he may smooth the bed of death, revive expiring life and counteract the depressing influence of those maladies which often disturb the tranquillity of the most resigned in their last moments. The life of a sick person can be shortened not only by the acts but also by the words or the manner of a physician. It is, therefore, a sacred duty to guard himself carefully in this respect, and to avoid all things which have a tendency to discourage the patient and to depress his spirits."

"A physician ought not to abandon a patient because the case is deemed incurable, for his attendance may continue to be highly useful to the patient and comforting to the relatives around him, even in the last period of a fatal malady, by alleviating pain and other symptoms, and by soothing mental anguish. To decline attendance, under such circumstances, would be sacrificing to fanciful delicacy and mistaken liberality, that moral duty which is independent of and far superior to all pecuniary consideration."

"Consultation should be promoted in difficult or protracted cases, as they give rise to confidence, energy and more enlarged views in practice."

"The opportunity which a physician not infrequently enjoys of promoting and strengthening the good resolutions of his patients, suffering under the consequences of vicious conduct ought never to be neglected. His counsels, or even his remonstrances, will give satisfaction, not offence, if they be proffered with politeness and evince a genuine love of virtue, accompanied by a sincere interest in the welfare of the person to whom they are addressed."

"There is no profession, from the members of which greater purity of character and a higher standard of moral excellence are required, than the medical, and to attain such eminence is a duty every physician owes alike to his profession and to his patients. It is due to the latter, as without it he cannot command their respect and confidence, and to both, because no scientific attainments can compensate for the want of correct moral principles. It is also incumbent upon the faculty to be temperate in all things, for the practice of physic requires the unremitting exercise of a clear and vigorous understanding, and on emergencies—for which no professional man should be unprepared—a steady hand, an acute eye and an unclouded head may be essential to the well-being and even to the life of a fellow-creature."

"Medicine is a liberal profession and those admitted into its ranks should found their expectations of practice upon the extent of their qualifications, not on intrigue or artifice."

"A physician in his intercourse with a patient under the care of another practitioner should observe the strictest caution and reserve. No meddling inquiries should be made, no disingenuous hints given relatives as to the nature and treatment of his disorder, nor any course of conduct pursued that may directly or indirectly tend to diminish the trust reposed in the physician employed."

"The same circumspection and reserve should be observed when, from motives of business or friendship, a physician is prompted to visit an individual who is under the direction of another practitioner. Indeed, such visits should be avoided, except under peculiar circumstances; and when they are made, no particular inquiries should be instituted relative to the nature of the disease or the remedies employed, but the topics of conversation should be as foreign to the case as circumstances will admit."

The spirit of the Code is tersely put in the Hippocratic oath. By this the practitioner of ancient times bound himself to enter his patient's house for the sole purpose of doing him good, and so to conduct himself as to avoid the very appearance of evil.

You have now heard the law of the State and the professional Code of Honor. Is there a higher law? Yes. "What is written in the law: how readest thou?"

"Thou shalt love the Lord thy God with all thy heart, and with all thy soul, and with all thy mind."

This is the first and greatest commandment, and the second is like unto it: "Thou shalt love thy neighbor as thyself."

This is the prelude to the parable of the good Samaritan who not only exemplified the law of love, but whose methods as a practitioner were nearly two thousand years in advance of the age.

The higher law then is the law of love, love for all mankind. If this spirit truly fills our hearts we will do our duty easily and naturally.

I will now ask your attention to some practical applications of the law, and I quote freely from the poet-physician, Dr. Oliver Wendell Holmes.*

*Medical Essays.

I warn you against all ambitious aspirations outside of the practice of medicine. It is often a disadvantage to a physician to be known for any accomplishment outside his profession. Medicine is the most difficult of sciences and the most laborious of arts. It will task all your powers of body and mind if you are faithful to it. Do not dabble in the muddy sewer of politics nor linger by the enchanted streams of literature, nor dig in the far off fields for the hidden waters of alien sciences.

The great practitioners are generally those who concentrate all their powers on their business. If there are here and there brilliant exceptions, it is only in virtue of extraordinary gifts and industry, to which few are equal.

Your patient has a right to the cream of your life, and not merely to the thin milk that is left after science has skimmed it off. *The best a physician can give is none too good for his patient.* The Code well says that a doctor owes it to his patient to be temperate. Intemperance in a physician partakes of the guilt of homicide, for the muddled brain may easily make a fatal blunder in a prescription and an unsteady hand transfix an artery in an operation.

There is a personal habit of less gravity than the one I have mentioned, which it is well to guard against. I mean the use of tobacco. We better than all men know how nicotine can, through its poisonous action on the nervous system, impair the sight, disorder the circulation and unsteady the hand. Besides, the sick are sensitive and nervous, and the fumes of tobacco may be disagreeable to many of them. The physician cannot be too particular to be nice in his personal habits, and if his presence is not suggestive of purity and cleanliness, his visits may be unwelcome. "Cleanliness is next to godliness"; and the want of it is sin.

Need I remind you of the duty of punctuality, and of the worry and distress to patients and their friends which the want of it occasions. Take pains to call on your patient as nearly as possible at the hour you have reason to think you are expected. It is a good rule. If you call too early my lady's hair may not be so smooth as could be wished, and if you keep her waiting too long her hair may be smooth but her temper otherwise.

It has been my habit always to get the weather gauge of my patient. I mean to place him so that the light falls on his face and not on mine. It is a kind of ocular duel that is about to take place between you; you are going to look through his features into his pulmonary and hepatic and other internal machinery, and he is going to look into yours quite as sharply to see what you think about his probabilities for time and eternity.

No matter how hard he stares at your countenance, he should never be able to read his fate in it. It should be cheerful as long as there is hope, and serene in its gravity when nothing is left but resignation. The face of a physician, like that of a diplomatist, should be impenetrable. Nature is a benevolent old hypocrite. She cheats the sick and the dying with illusions, better than any anodynes. If there are cogent reasons why a patient should be undeceived, do it deliberately and advisedly; but do not betray your apprehensions through your tell-tale features.

Your patient has no more right to all the truth you know than he has to the medicines in your saddle-bags, if you carry that kind of cartridge-box for the ammunition that slays disease. He should get only so much as is good for him. It is a terrible thing to take away hope from a fellow creature. Be very careful what names you let fall before your patient. He knows what it means when you tell him he has tubercles or Bright's disease, and, if he hears the word carcinoma, he will certainly look it out in a medical dictionary, if he does not interpret its dread significance on the instant.

I advise you then, as far as possible, keep your doubts to yourself, and give the patient the benefit of your decision. Firmness, gentle firmness, is absolutely necessary in this and certain other relations.

If you cannot acquire and keep the confidence of your patient, it is time for you to give place to some other practitioner who can. If you are wise and diligent you can establish relations, which they will find it hard to break. But if they wish to employ another person, who, as they think, knows more than you do, do not take it as a personal wrong. A patient believes another man can restore him to health, which as he thinks you have not the skill to do. No matter whether the patient is right or wrong, it is a great impertinence to think you have any property in him. Your estimate of your own ability is not the question; it is what the patient thinks of it. Remember, too, not to feel aggrieved against the brother physician who succeeds you in the case. A patient has a right to change his physician as often as he pleases, he is only bound to do it in the right way; that is, to discharge the one, before he engages the other.

Be careful in regard to the use of drugs. I hold that it is a good rule not to prescribe any drug unless you can give a reason for it. If your patient *will* have a prescription when he does not need it, indulge his fancy with a placebo. A doctor who relies altogether on drugs is not the master but the slave of his profession.

Remember that in many cases medication is entirely secondary, and your patient's health may depend on the diet, regimen, temperance and mode of life; or change of climate; in short on his environments and his relation thereto. You will, if you are wise, look into these matters which prescriptions cannot cover.

Make it a rule to look carefully over every prescription before it leaves your hands. This may save you from some day being the author of a homicidal document, which, if not intercepted by a druggist more careful than you, might cause you lasting remorse and perhaps loss of reputation.

I beg now to call your attention to a few points in which I think the profession sometimes fails to come up to the full measure of its duty to the patient.

I have often noted a want of due care in the administration of general anesthetics. The administrator should never lose sight of the fact, that the life of the patient is for the time being entirely in his hands, and is dependent for minutes or hours upon the skill and attention which he gives to the performance of his duties. If he is faithful he will watch the patient, and not the operation. He will not beguile the time, as I have often noticed, with interesting stories; nor allow others to do so. He will at every moment be aware of the exact condition of his patient, especially his respiration. His conduct should be such that the operator can devote his whole attention to the operation in hand and have his mind free from anxiety in regard to other matters.

I believe that the majority of the deaths under anesthesia are due to carelessness and lack of that unremitting attention which is the first and last duty of the administrator. He must "watch as if on that alone hung the issue of the hour."

I think some members of the profession hold mistaken views in regard to the administration of chloroform in cases of labor. They either decline, or give it so sparingly that while keeping the word of promise to the ear, they break it to the hope of the sufferer. I think chloroform should be used in all labor cases where the degree of suffering warrants it.

To what extent it should be given is an important matter, and will depend upon the circumstances of the case. In ordinary cases it should be given in quantity sufficient only to deaden pain, and when this point is reached the patient will be unable to ask for more. Given to this extent it does not retard labor as many suppose, but accelerates it; the removal of the pain calling into full play the voluntary muscles. In cases so rapid that laceration is imminent, chloroform can be given to the extent of controlling the involuntary muscles also; thus reducing the pains within the limit of safety.

My obstetric practice has been singularly free from accidents and complications; such as protracted labor, retained placenta, post-partum hemorrhage and fevers. I cannot attribute this to any other cause than to the regular use of chloroform, on the principles above indicated. So far as the dangers are concerned I have seen none, and I have heard that Sir James Simpson gave chloroform in twelve thousand cases of labor without an accident.

I must warn you against the indiscriminate use of bandages and surgeon's plaster in the treatment of injuries. I have seen these articles so tightly applied as not only to cause great suffering, but sometimes even to endanger a limb. The edges of a wound can seldom be properly approximated by plaster, and a limb should not be bandaged simply because it is broken. It is seldom necessary or safe to apply a bandage directly to the skin, and it should never be done without a reason. Tho proper function of a bandage is to retain splints and other apparatus in position, and it should be applied no tighter than is necessary to affect this purpose. If the surgeon bears this in mind he will not only contribute much to the comfort of his patient, but perhaps will avoid the risk of inflicting serious injury.

Now last, but not least, the physician's duty to his patient requires that he shall keep him *free from pain*, as far as is consistent with the proper treatment of the case. So far as I can judge, many members of the profession, even some of its brightest exemplars, are strangely wanting in their duty in this regard. I am satisfied that this is due more to an unwarranted dread of the evil consequences of narcotics and anodynes, than to a want of feeling. The error has sometimes been committed through a desire, laudable enough, to hasten through a duty difficult to a surgeon and painful to the patient. The relief of suffering requires time and trouble, and a minor operation is often hurried through, at the cost of the patient's feelings. I may remark that when a physician happens to be the patient I have never noticed that there was any lack of preparation for the prevention of pain.

But it may be asked by what right I speak thus to experts, who are themselves qualified to judge what is best for their own cases. I can only say, that this question has interested me especially from the beginning of my professional life, and my official position has made it my duty to supervise the work of some of my professional brethren. Moreover, I feel that if I can convince any of you of the correctness of the views I so strongly hold, I will have done something towards lessening human suffering.

I am satisfied from many trials, and repeated observations, that injured persons rally more promptly from a shock and recover more quickly from their injuries, if they are kept as free as possible from pain. In most cases anodynes and narcotics are indicated for a short time only; the shorter, if they are promptly and boldly used up to the proper limit. This is marked by the relief of pain. If the limit is exceeded, the evil effects of the drug will be shown in disorders of the digestion, and locking up the secretions. The danger of narcotism can be avoided by the caution, *never to wake a patient for the administration of a dose.*

Whenever it is practicable, I prefer the hypodermic method of administration because it is more efficient, less injurious, and, above all, is in the control of the physician.

I wish to be distinctly understood as maintaining that the prompt and judicious use of opiates not only relieves suffering, but by calming the mind, equalizing the circulation, and making the patient indifferent to his troubles, has a directly curative power. This is more true in acute than in chronic cases; but sometimes even in these, the effect in breaking up a vicious chain of symptoms is marvelous. Yet I do not recommend the continued use of narcotics in chronic cases unless they are hopeless or malignant. I have for years used opiates freely in my practice, but I have never known a patient to acquire the habit therefrom.

Such then, my professional brethren, is the measure of our responsibility. The standard is high, but that should only increase our zeal to attain it. If we regard our patients as men and brethren, we will, without the restraint of law and code, follow the golden rule and do unto others as we would have them do unto us. If we are faithful to this, what is our reward? Not only "the promise of the life that now is," but when the Son of Man, who was our brother here on earth, shall be the KING upon the throne of his glory, he will say, "Inasmuch as ye have done it unto one of the least of these my brethren, ye have done it unto ME."

